# Connectivity, Pathology, and ApoE4 Interactions Predict Longitudinal Tau Spatial Progression and Memory

**DOI:** 10.1002/hbm.70083

**Published:** 2024-12-09

**Authors:** Jacob Ziontz, Theresa M. Harrison, Corrina Fonseca, Joseph Giorgio, Feng Han, JiaQie Lee, William J. Jagust, Alzheimer's Disease Neuroimaging Initiative

**Affiliations:** ^1^ Department of Neuroscience UC Berkeley Berkeley California USA; ^2^ School of Psychological Sciences, College of Engineering, Science and the Environment, University of Newcastle Newcastle New South Wales Australia; ^3^ Lawrence Berkeley National Laboratory Berkeley California USA

**Keywords:** aging, Alzheimer's, functional connectivity, longitudinal, tau pathology

## Abstract

Tau pathology spread into neocortex indicates a transition from healthy aging to Alzheimer's disease (AD). Connectivity between tau epicenters and later accumulating regions of cortex has been proposed as a mechanism of tau spread, but how this relationship changes with greater AD pathology burden or genotype is not understood. We investigated tau accumulation in two key regions, precuneus and inferior temporal cortex, using resting state functional connectivity (rsFC) and longitudinal PET imaging from a multicohort sample of cognitively unimpaired older adults. We examined how baseline tau PET, Aβ PET, and ApoE4 genotype status interact with rsFC between hippocampus and these downstream regions to predict rate of tau accumulation in neocortex. We found that the 3‐way interaction between connectivity, baseline tau, and baseline Aβ or ApoE4 status was associated with neocortical tau accumulation in precuneus and inferior temporal cortex. In addition, baseline tau, Aβ, and ApoE4 status also moderated the association between connectivity and rate of memory decline. Together, these results suggest that the extent and distribution of future tau accumulation may be predicted by the interaction of baseline connectivity, AD pathology, and genetic risk.

## Graphical abstract

Baseline Alzheimer's disease pathology interacts with individualized functional connectivity between hippocampus and vulnerable areas of neocortex to predict rate of accumulation in tau pathology in these downstream regions. Spatiotemporal tau progression and its consequences for cognitive function are thus influenced by interactions between baseline pathology and functional connectivity.

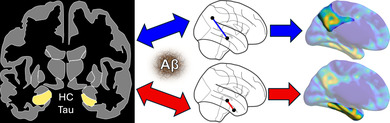

## 1 Introduction

Hyperphosphorylated tau protein and fibrillar beta‐amyloid (Aβ) are the hallmark neuropathologies of Alzheimer's disease, with neurofibrillary tangles of tau accumulating both intra‐ and extracellularly and Aβ plaques largely found in the extracellular space (Jack et al. 2018a). Aβ accumulates in a diffuse manner across the neocortex, but tau pathology has a stereotyped pattern of deposition, originating in the medial temporal lobe before accumulating in proximal areas of the temporal and medial parietal cortices (Braak and Braak 1991). Tau pathology is found in the medial temporal lobe (MTL) of most older adults, even those without cognitive impairment (Crary et al. 2014), but its accumulation in other areas of the cortex is thought to represent a transition to Alzheimer's disease and is associated with neurodegeneration and cognitive decline (Aschenbrenner et al. 2018; Iaccarino et al. 2018). A variety of evidence suggests that substantial tau deposition outside of MTL occurs only with the presence of Aβ pathology, which is likely a key component of this process (Bennett et al. 2017; He et al. 2018). However, the mechanisms of tau accumulation and its transition out of the MTL are poorly understood.

Though the spread of tau pathology in Alzheimer's disease does not always follow a single homogeneous trajectory (Vogel et al. 2021), in sporadic AD tau spread outside of medial temporal lobe predominantly occurs in inferior and lateral regions of the temporal cortex, as well as frequently in medial parietal regions such as the retrosplenial cortex and precuneus (Schöll et al. 2016; Johnson et al. 2016; Harrison et al. 2019; Sanchez et al. 2021). In particular, the inferior temporal cortex (IT) has been proposed as a key region of tau spread, with pathology in this region representing a transition to mild cognitive impairment (MCI) and AD (Lee et al. 2022). Indeed, several studies have identified inferior temporal tau accumulation as indicative of future atrophy, cognitive impairment, and functional decline (Scott et al. 2020; Halawa et al. 2019; Sperling et al. 2019). Medial parietal tau has also been observed to be an area of relatively early tau accumulation (Harrison et al. 2019; Jack et al. 2018b; Maass et al. 2017), and substantial increase in accumulation in this region is observed in MCI and AD (Maass et al. 2019). This region is also notable as a site of early Aβ deposition and subsequent network disruption (Sperling et al. 2009), and thus the pathway between medial temporal lobe structures such as hippocampus and medial parietal regions may be of particular importance for the interaction of tau and Aβ pathologies. Furthermore, following the proposed role of anterior temporal brain regions in object/item memory and posterior medial region in scene/context memory (Ranganath and Ritchey 2012; Ritchey, Libby, and Ranganath 2015; Epstein et al. 2017), there may be distinct cognitive consequences for tau accumulation in IT and medial parietal cortices. As tau spreads from its origin in medial temporal lobe to these regions, likely along distinct neuronal pathways, disruptions in neuronal signaling and neurodegeneration may lead to differentiable declines in cognitive function over time (Harris et al. 2020; Ossenkoppele et al. 2022; Hanseeuw et al. 2019).

Considerable evidence from in vitro studies (Pooler et al. 2013), animal models (Wu et al. 2016; Ahmed et al. 2014), and human neuroimaging investigation (Vogel et al. 2020; Cope et al. 2018) suggests that tau pathology spreads throughout the cortex in a transsynaptic manner. Tau has been observed to propagate via the extracellular space from one neuron to another, and the release of tau is enhanced by neuronal activity, increasing tau pathology accumulation in vivo (Pooler et al. 2013; Wu et al. 2016; Ahmed et al. 2014). The spread of tau pathology throughout the brain has been corroborated by analysis of functional neuroimaging measures in older adults and individuals with Alzheimer's disease. In particular, resting state functional magnetic resonance imaging (rsfMRI) has been used as a way to model the spread of tau throughout the cortex (Adams et al. 2019; Franzmeier et al. 2019). This work is based in the principle that functionally connected brain regions, i.e., areas of the brain that show correlated blood oxygen level‐dependent (BOLD) signal over time, may represent pathways between coactive regions that facilitate the transsynaptic spread of tau pathology. Early graph theoretical analysis of rsfMRI functional connectivity in Alzheimer's disease indicated that more positively correlated (i.e., strongly connected) nodes exhibit greater tau pathology burden, favoring the notion of transneuronal tau spread rather than tau arising in areas of high metabolic demand (Cope et al. 2018). More recently, converging evidence has shown that functional connectivity strength between tau epicenters and other areas of cortex correlates with the degree and covariance of tau pathology in these downstream areas (Adams et al. 2019; Franzmeier et al. 2019; Ziontz et al. 2021). Resting state functional connectivity has also been used to model future tau accumulation (Vogel et al. 2020) and baseline functional connectivity has been shown to associate with rate of tau accumulation in individuals across the AD continnuum (Franzmeier et al. 2020a, 2020b; Therriault et al. 2022), emphasizing the critical role of functional connectivity in AD‐phenotype patterns of tau accumulation across the neocortex. However, little attention has been given to the factors that drive tau pathology spread along the pathways of earliest accumulation in cognitively unimpaired individuals.

Functional pathways in the brain have been proposed as the conduits along which neurodegenerative pathologies tend to spread (Vogel et al. 2023), but additional factors are needed to explain the variability in extent and distribution of pathology across individuals, particularly at the earliest stages of accumulation. If tau spreads to downstream neocortical areas along pathways of functional connectivity, the degree of tau pathology in upstream medial temporal lobe regions should influence the rate of this spread. Indeed, an increased rate of tau spread into the neocortex is associated with greater baseline medial temporal tau pathology and global Aβ burden (Sanchez et al. 2021). Furthermore, the presence of cortical Aβ has been associated with hyperexcitability (Targa Dias Anastacio, Matosin, and Ooi 2022) and changes in network connectivity (Ossenkoppele et al. 2019), and maybe a critical factor in driving medial temporal lobe excitability and tau accumulation (Giorgio et al. 2024). In addition, carriers of the apolipoprotein E (ApoE) ε4 allele have exhibited hyperexcitability in medial temporal lobe structures such as hippocampus (Koutsodendris et al. 2023; Nuriel et al. 2017), as well as differences in the connectivity of functional brain networks including default mode and temporal networks (Filippini et al. 2009; Cacciaglia et al. 2023). ApoE4 status has also been linked to greater rates of tau accumulation (Baek et al. 2020; Buckley et al. 2019; Young et al. 2023), and recent evidence has suggested that Aβ may be a critical mediating factor in the relationship between ApoE genotype and connectivity‐mediated tau spreading (Steward et al. 2023). Taken as a whole, high functional connectivity between areas of early pathology accumulation, modulated by greater baseline tau, Aβ, and/or ApoE genotype, maybe the conditions under which tau accumulates most quickly, and therefore represent a useful framework for predicting future regional tau accumulation at the earliest stages of disease. However, previous work has not fully addressed how individual functional connectivity strength influences the rate of downstream tau accumulation, nor how these factors interact to predict changes in cognitive function that may ensue.

In the present study, we used rsfMRI and longitudinal PET imaging to investigate the spread of tau pathology from medial temporal lobe to neocortex in a multicohort sample of cognitively unimpaired older adults. For each individual, we measured resting state functional connectivity between hippocampus and both inferior temporal cortex and precuneus, two key potential pathways of neocortical tau accumulation (Sanchez et al. 2021; Lee et al. 2022; Ziontz et al. 2021; Jacobs et al. 2018). We then assessed the relationship between rate of longitudinal tau accumulation in these regions and interactions between baseline pathology and functional connectivity, i.e., the 3‐way interaction between baseline connectivity strength, tau pathology, and cortical Aβ. We further tested whether ApoE genotype in these same individuals modulated this tau‐connectivity relationship. Finally, we examined the relationship between pathology‐connectivity interaction and decline in memory performance over time. We hypothesized that the relationship between higher functional connectivity and downstream tau accumulation would be modulated by baseline tau and Aβ pathology, as well as ApoE genotype, to show the strongest relationships with downstream tau accumulation. In addition, we hypothesized that the interaction of these factors would be associated with a decline in memory performance.

## 2 Results

### 2.1 Baseline Tau, Aβ, and Functional Connectivity Interaction Is Associated With Longitudinal Tau

**FIGURE 1:**
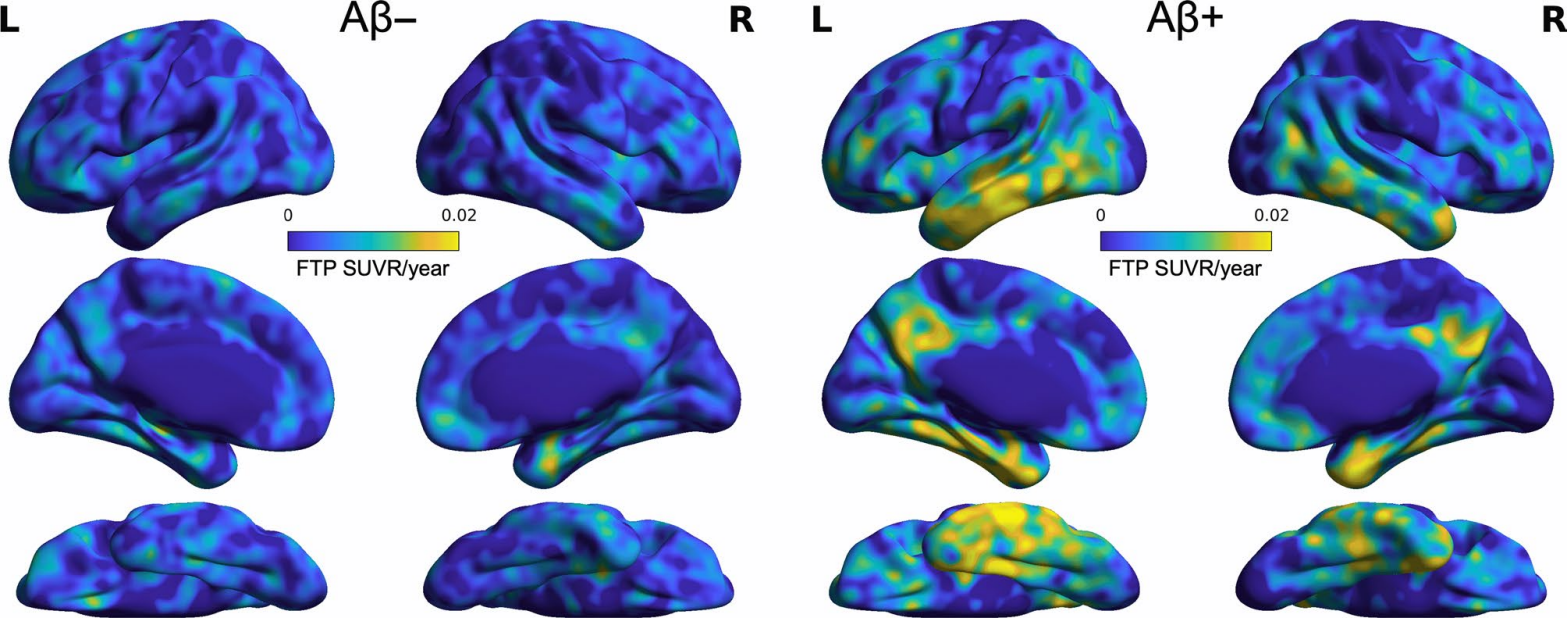
Voxelwise tau accumulation in combined sample of cognitively unimpaired older adults. Longitudinal rate of tau accumulation was computed using a simple linear regression model for all flortaucipir (FTP) PET scans for each cognitively unimpaired (CU) individual in the combined sample of BACS and ADNI participants. Group average voxelwise FTP slopes (SUVR/year) are plotted for Aβ− (left) and Aβ+ (right) subjects.

To investigate the association between functional connectivity, baseline pathology, and longitudinal rate of tau accumulation, we utilized a sample of 110 cognitively unimpaired older adults (Table 1), *n* = 67 individuals from the Berkeley Aging Cohort Study (BACS), and *n* = 43 individuals from the Alzheimer's Disease Neuroimaging Initiative (ADNI). Participants were between 61 and 93 years of age (*M* = 76.4 years, SD = 6.4 years) cognitively unimpaired at baseline with an interval between 3 T rsfMRI and flortaucipir (FTP) PET scans of no greater than 1 year (*M* = 45 days, SD = 52 days). Participants also had a concurrent Aβ PET scan, and at least 2 FTP PET time points. A subset of 106 individuals had genotype data available to determine ApoE ε4 status. There were no significant differences in demographics between participants from the BACS and ADNI cohorts (Table S1), though ADNI individuals had a shorter interval between baseline rsfMRI scan and FTP PET scan (*p* = 0.02). For all participants, resting state functional connectivity between native space Desikan–Killiany atlas regions was computed, and functional connectivity values were normalized within cohorts to be able to account for acquisition differences when comparing these values between BACS and ADNI (see Methods). In addition, we computed the FTP slope using linear regression for each subject in each voxel of the brain. Visualizing this average voxelwise longitudinal rate of change, tau accumulation over time was observed primarily in lateral and inferior temporal cortices as well as medial parietal areas bilaterally and was visibly higher in Aβ+ compared with Aβ− individuals (Figure 1).

**TABLE 1:** Demographics of combined BACS and ADNI sample.

CU older adults			
	All (*n* = 110)	Aβ− (*n* = 58)	Aβ+ (*n* = 52)
	Mean (SD)		
Age (years)	76.4 (6.4)	74.4 (7.0)	78.7 (4.8)
Centiloids	28.0 (38.5)	3.1 (6.5)	55.7 (40.5)
Meta ROI tau slope (SUVR/year)	0.02 (0.01)	0.01 (0.01)	0.03 (0.01)
rsfMRI‐tau interval (days)	45.0 (52.0)	52.1 (53.6)	37.1 (49.4)
# FTP PET scans	2.81 (0.88)	2.79 (0.83)	2.81 (0.94)

	*n* (%)		
Sex (female)	67 (60.9%)	33 (56.9)	34 (65.4%)
ApoE𝜀4+	35 (33.0%)	9 (16.0%)	26 (52%)

Abbreviations: CU, cognitively unimpaired; FTP, flortaucipir; PET, positron emission tomography; rsfMRI, resting‐state functional magnetic resonance imaging; SUVR, standard uptake value ratio.

**FIGURE 2:**
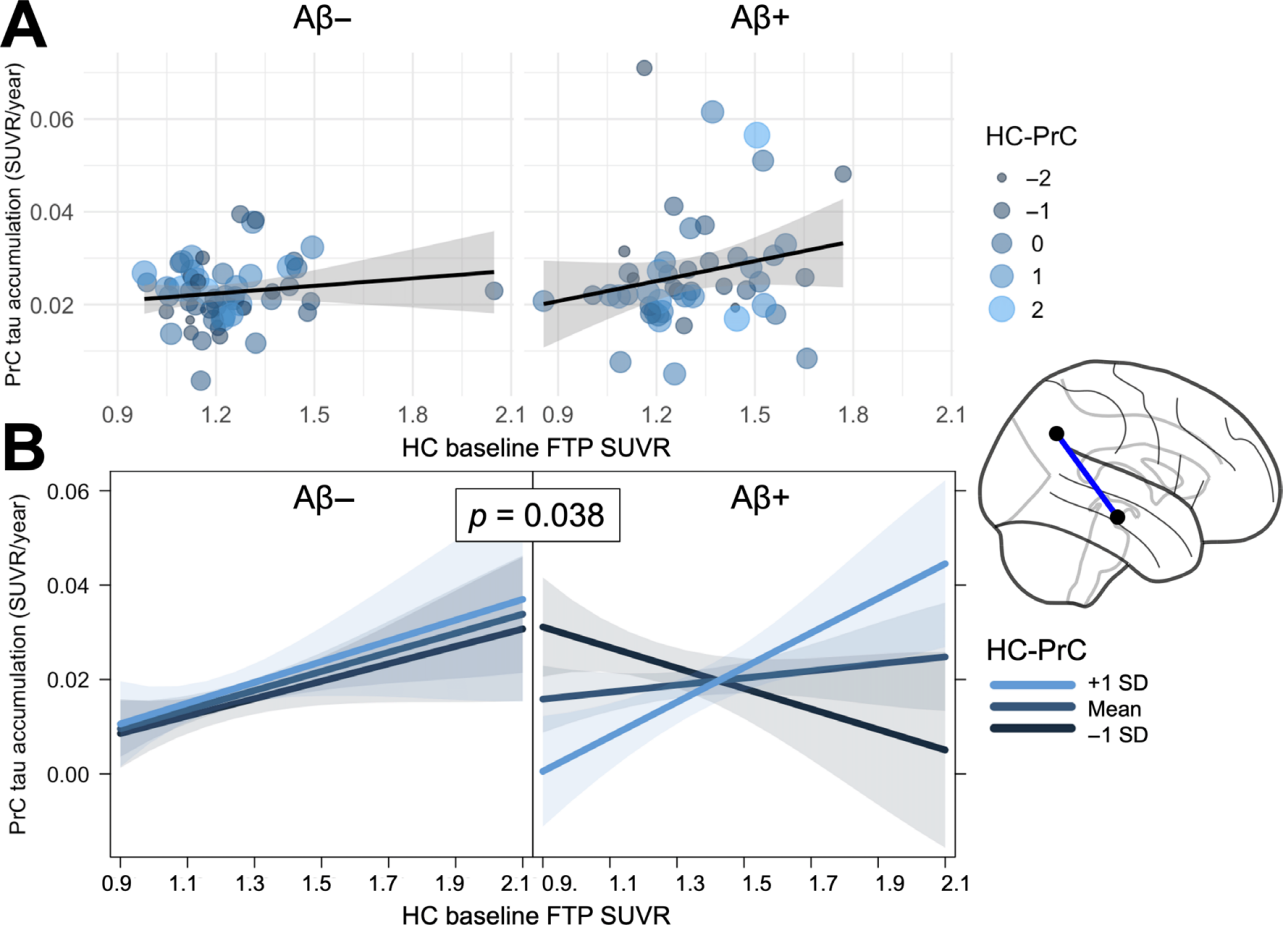
Hippocampus–precuneus pathology‐connectivity interaction is associated with precuneus tau accumulation. (A) Rate of tau accumulation in the precuneus (PrC) as a function of baseline hippocampal (HC) tau signal. Points represent a single Aβ− (*left*) or Aβ + (*right*) subject, with size and color corresponding to the strength of functional connectivity between hippocampus and precuneus (HC‐PrC). (B) From a linear model adjusting for age, sex, and choroid plexus FTP signal, visualization of association between precuneus rate of tau accumulation and the 3‐way interaction of baseline HC tau, Centiloids, and HC‐PrC functional connectivity. Lines represent predicted association for 3 different levels of HC‐PrC connectivity. Panels visualize predicted associations at the mean Centiloid value of Aβ− (*left*) and Aβ + (*right*) participants.

We next investigated the association between the rate of tau pathology accumulation and functional connectivity in 2 regions of interest: precuneus (PrC) and inferior temporal cortex (IT). Partial‐volume corrected (PVC) rate of tau accumulation in each region was first calculated using linear mixed‐effects models with time from baseline as the only predictor and random effects of slope and intercept. These tau accumulation rates were then used as the dependent variable in linear mixed effects models testing the 3‐way interaction (and all related 2‐way interactions and main effects) between hippocampus–precuneus resting state functional connectivity (HC‐PrC), baseline hippocampal tau, and baseline cortical Aβ burden (Centiloids), with additional fixed effects for age, sex, and choroid plexus FTP SUVR and a random effect of cohort. We found a main effect of greater baseline HC tau on the rate of tau accumulation in the downstream precuneus (*β* = 0.014, *p* = 0.025), and further observed a 2‐way interaction between HC‐PrC and baseline HC tau, such that there was a closer association between HC tau and precuneus tau accumulation for stronger HC‐PrC connectivity (*β* = 0.016, *p* = 0.016). Critically, we also observed a 3‐way interaction between HC‐PrC, baseline HC tau, and baseline Centiloids (*β* = 0.006, *p* = 0.038), indicating that the strongest association between HC tau and precuneus tau accumulation was present in individuals with greater HC‐PrC connectivity and greater baseline Aβ burden (Figure 2). This 3‐way interaction was also significant in the BACS and ADNI cohorts separately (Figure S1). To test the specificity of these pathology‐connectivity interactions along this hypothesized hippocampus–precuneus pathway, we repeated these analyses using parahippocampal gyrus as a control upstream medial temporal lobe region and did not find that precuneus tau accumulation was related to the 3‐way interaction between baseline parahippocampal‐precuneus connectivity, parahippocampal tau, and Centiloids (*β* = 0.00002, *p* = 0.90). Examining the superior parietal cortex as a control downstream neocortical region, we did not find that tau accumulation in this region was related to the 3‐way interaction between hippocampus‐superior parietal connectivity, hippocampal tau, and Centiloids (*β* = 0.0001, *p* = 0.41).

**FIGURE 3:**
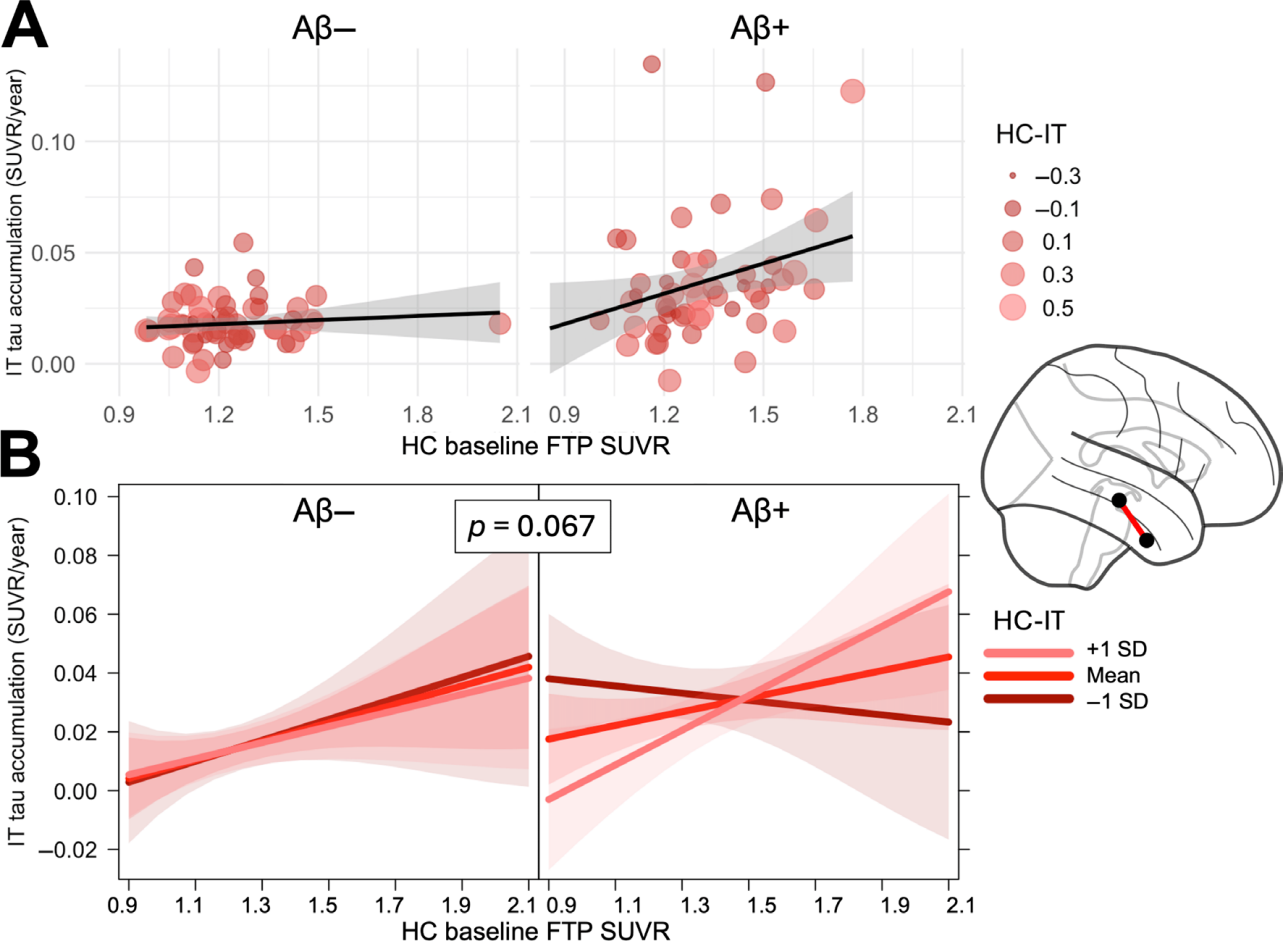
Hippocampus‐inferior temporal pathology‐connectivity interaction is associated with inferior temporal tau accumulation. (A) Rate of tau accumulation in inferior temporal (IT) cortex as a function of baseline hippocampal (HC) tau signal. Points represents a single Aβ− (*left*) or Aβ + (*right*) subject, with size and color corresponding to strength of functional connectivity between hippocampus and inferior temporal cortex (HC‐IT). (B) From a linear model adjusting for age, sex, and choroid plexus FTP signal, visualization of association between precuneus rate of tau accumulation and the 3‐way interaction of baseline HC tau, Centiloids, and HC‐IT functional connectivity. Lines represent predicted association for 3 different levels of HC‐IT connectivity. Panels visualize predicted associations at mean Centiloid value of Aβ− (*left*) and Aβ + (*right*) participants.

Similarly, we examined the association between the rate of tau accumulation in IT and the 3‐way interaction between hippocampus‐inferior temporal resting state functional connectivity (HC‐IT), baseline hippocampal tau, and baseline cortical Aβ. We found a main effect of greater baseline HC tau on the rate of tau accumulation in IT (*β* = 0.028, *p* = 0.049), though the 2‐way interaction between HC‐IT and baseline HC tau did not reach significance (*β* = 0.016, *p* = 0.23). The 3‐way interaction between HC‐IT, baseline HC tau, and baseline Centiloids was associated with IT tau accumulation (*β* = 0.007, *p* = 0.067), suggesting a trend towards the relationship with HC‐IT connectivity being modulated by baseline tau and Aβ pathology (Figure 3). Modeling these relationships in each cohort separately, this 3‐way interaction was significantly associated with IT tau accumulation in ADNI, but not in BACS (Figure S2). Again, we repeated these analyses using parahippocampal gyrus as a control upstream medial temporal lobe region and did not find that inferior temporal tau accumulation was related to the 3‐way interaction between baseline parahippocampal‐inferior temporal connectivity, parahippocampal tau, and Centiloids (*β* = −0.0001, *p* = 0.64). Examining the superior temporal cortex as a control downstream neocortical region, we did not find that tau accumulation in this region was related to the 3‐way interaction between hippocampus‐superior temporal connectivity, hippocampal tau, and Centiloids (*β* = −0.00006, *p* = 0.83).

### 2.2 ApoEε4 Genotype Modulates Connectivity Association With Tau Accumulation

**FIGURE 4:**
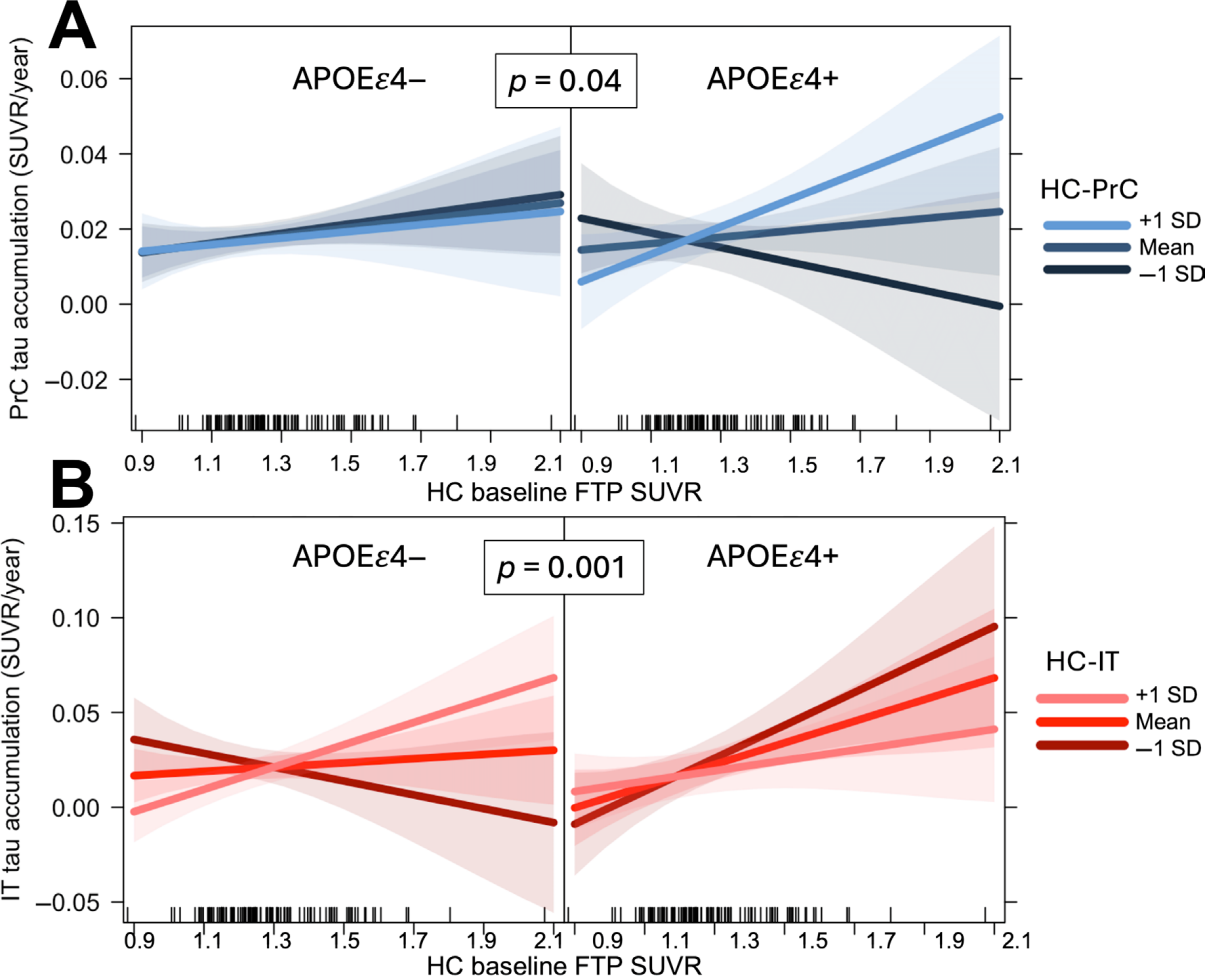
Connectivity modulated by ApoEε4 genotype is associated with rate of tau accumulation. (A) Visualization of 3‐way interaction between hippocampus–precuneus functional connectivity (HC‐PrC), baseline HC tau, and *ApoEε4+* status. From a linear model adjusting for age, sex, and choroid plexus FTP signal, association between HC baseline tau is modulated by HC‐PrC and *ApoEε4* positivity. (B) Visualization of 3‐way interaction between hippocampus‐inferior temporal functional connectivity (HC‐IT), baseline HC tau, and *ApoEε4+* status. From a linear model adjusting for age, sex, and choroid plexus FTP signal, association between HC baseline tau is modulated by HC‐IT and *ApoEε4* negativity. Lines represent predicted association for 3 different levels of functional connectivity, and vertical ticks along the x‐axis visualize individual HC baseline tau values. Panels visualize predicted associations for *ApoEε4‐* (*left*) and *ApoEε4+* (*right*) participants.

We next tested if there were dissociable effects of ApoE ε4 status and Aβ on the relationship between baseline tau, connectivity, and longitudinal tau accumulation. We again used linear mixed effects modeling with a random effect of cohort to assess the relationship between precuneus tau accumulation and the 3‐way interaction between ApoE ε4 carrier status, baseline HC tau, and HC‐PrC connectivity. We did not observe any significant main effects or 2‐way interactions, but the 3‐way interaction was associated with precuneus tau accumulation (*β* = 0.032, *p* = 0.04), such that the strongest association between baseline HC tau and precuneus tau accumulation tended to be present in ApoE ε4+ individuals with greatest HC‐PrC connectivity (Figure 4a). This 3‐way interaction remained significantly associated with precuneus tau accumulation in a separate model adjusting for the main effect of baseline Centiloids (*β* = 0.034, *p* = 0.028). In analyses separated by cohort, the interaction was significantly associated with PrC tau accumulation in BACS, but not in ADNI (Figure S3). Next, in a model predicting IT accumulation, we found a significant 3‐way interaction between ApoE ε4 carrier status, baseline tau, and HC‐IT connectivity (*β* = −0.087, *p* = 0.001), indicating that HC‐IT connectivity tended to modulate the association between HC baseline tau and inferior temporal tau accumulation specifically in ApoE ε4− individuals (Figure 4b). This interaction remained significantly associated with IT tau accumulation in a separate model with baseline Centiloids as a main effect (*β* = −0.083, *p* = 0.001), but not when analyzed in each cohort independently (Figure S4).

### 2.3 Pathology‐Connectivity Interactions Are Linked to Memory Decline

Finally, we investigated whether pathology‐connectivity interactions were also related to decline in episodic memory, a critical downstream consequence of tau pathology accumulation. For this analysis, we focused on the subsample of 67 BACS participants with 546 longitudinal neuropsychological testing time points spanning up to 18 years (*M* = 8.9, SD = 4.0) and up to 15 cognitive time points per individual (*M* = 8.1, SD = 3.3). Similar to rate of tau accumulation, rate of memory decline was calculated using linear mixed effects models with time from baseline as the only fixed predictor and random effects of slope and intercept. Given the proposed role of the medial parietal cortex in spatial processing and memory (Epstein et al. 2017), we examined the relationship between pathology‐connectivity interactions and a visuospatial memory composite of Visual Reproduction I and II performance, as well as a verbal memory composite of the California Verbal Learning Test (CVLT) short‐ and long‐delay‐free recall components.

**FIGURE 5:**
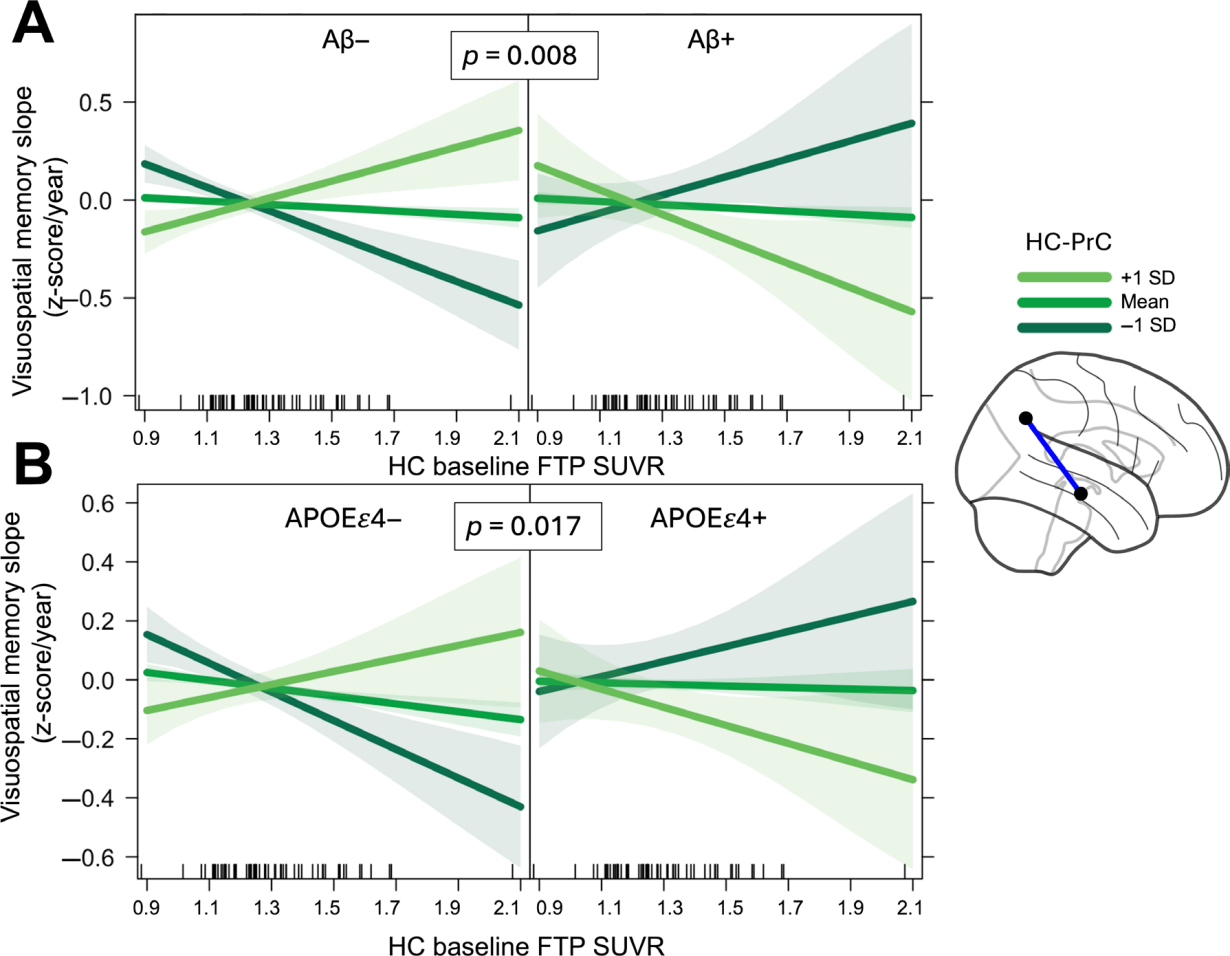
HC‐PrC pathology‐connectivity interaction is associated with visuospatial memory decline. (A) Visualization of 3‐way interaction between hippocampus–precuneus functional connectivity (HC‐PrC), baseline HC tau, and Centiloids. Adjusting for age, sex, and choroid plexus FTP signal, association between HC baseline tau and rate of decline is visuospatial memory performance is modulated by HC‐PrC and Centiloids. Panels visualize predicted associations at mean Centiloid value of Aβ− (*left*) and Aβ + (*right*) participants. (B) Visualization of 3‐way interaction between hippocampus–precuneus functional connectivity (HC‐PrC), baseline HC tau, and *APOEε4* status. Adjusting for age, sex, and choroid plexus FTP signal, association between HC baseline tau and rate of decline is visuospatial memory performance is modulated by HC‐PrC and *APOEε4* positivity. Lines represent predicted association for 3 different levels of functional connectivity, and vertical ticks along the x‐axis visualize individual HC baseline tau values. Panels visualize predicted associations for *APOEε4−* (*left*) and *APOEε4+* (*right*) participants.

We first assessed the association between rate of visuospatial memory decline and pathology‐connectivity interactions at baseline using linear regression models adjusting for age, sex, and choroid plexus FTP SUVR. We observed a main effect of baseline HC tau on rate of visuospatial memory decline (*β* = −0.08, *p* = 0.01), as well as a 2‐way interaction of HC‐PrC and Centiloids (*β* = −0.34, *p* = 0.03) indicating that greater HC‐PrC connectivity was associated with less steep memory decline at lower baseline Centiloids. Critically, we also observed a significant 3‐way interaction between baseline HC‐PrC connectivity, HC tau, and Centiloids (*β* = −3.97, *p* = 0.008), such that the greatest decline in visuospatial memory performance occurred in individuals with highest baseline Aβ, HC‐PrC connectivity, and HC tau (Figure 5a). We repeated this analysis examining the interaction between baseline connectivity, pathology, and ApoE genotype and including a main effect of Centiloids in the linear regression model, and again found a significant 3‐way interaction between baseline HC‐PrC connectivity, HC tau, and ApoE genotype (*β* = −0.68, *p* = 0.017), such that the greatest decline in visuospatial memory performance was observed in ApoE ε4+ individuals with greater HC‐PrC connectivity and greater HC tau (Figure 5b). We replicated this analysis using HC‐IT connectivity in place of HC‐PrC but did not observe any significant pathology‐connectivity interactions associated with decline in visuospatial memory performance.

In assessing rate of change in verbal memory performance, we observed a main effect of baseline HC tau on verbal memory decline (*β* = −0.05, *p* = 0.04), and again observed a 2‐way interaction of HC‐PrC and Centiloids (*β* = −0.29, *p* = 0.01). The 3‐way interaction between baseline HC‐PrC connectivity, HC tau, and Centiloids was not significant (*β* = −1.99, *p* = 0.07), but suggested a trend toward individuals with highest baseline Aβ, HC‐PrC connectivity, and HC tau exhibiting the steepest verbal memory decline (Figure S5). Again, we repeated this analysis examining the interaction between baseline connectivity, pathology, and ApoE genotype and including a main effect of Centiloids in the linear regression model, but did not find a significant 3‐way interaction (*β* = −0.10, *p* = 0.66). Finally, we again carried out these analyses using HC‐IT connectivity in place of HC‐PrC, but did not observe any significant pathology‐connectivity interactions associated with decline in verbal memory performance.

## 3 Discussion

This study utilized rsfMRI, longitudinal PET imaging, and longitudinal cognitive performance data to investigate the factors involved in the earliest tau accumulation in cognitively unimpaired older adults. We found that the interaction of Aβ pathology and/or ApoE genotype, baseline tau pathology, and functional connectivity between hippocampus and key regions of downstream tau accumulation was associated with the fastest increases in tau pathology in these regions. These pathology‐connectivity interactions were further associated with domain‐specific decline in cognitive function in these unimpaired individuals. Taken together, these results suggest that the strength of connectivity between hippocampus and downstream regions is modulated by the degree of baseline AD pathology to predict tau accumulation, and the interactions between these factors have the potential to explain differences in rates and distribution of tau accumulation at the earliest stages of cognitive impairment.

A particularly intriguing finding motivating the joint study of network connectivity and neurodegenerative disease is that patterns of regional brain atrophy tend to match the topography of functional and structural networks in healthy individuals (Seeley et al. 2009). This insight has led to the investigation of functional connectivity in aging and Alzheimer's disease as a potential mechanism that drives the spread of pathology throughout the brain, with corresponding consequences for regional brain atrophy and domain‐specific cognitive decline. From the cortical origin of tau in the transentorhinal region, tau may spread along pathways of strong functional connectivity first to hippocampus (Ziontz et al. 2021) and subsequently to other areas of cortex (Adams et al. 2019) known to accumulate pathology and leading to neurodegeneration later in disease progression (La Joie et al. 2020). Our results support this view, showing that the association between tau accumulation and functional connectivity between hippocampus and both precuneus and inferior temporal cortex in part depends on the degree of baseline AD pathology. We found this relationship to be somewhat more robust for the precuneus than for inferior temporal cortex, which may reflect differences in fMRI signal in these regions, as inferior temporal areas tend to have a relatively high degree of signal disruption (Deichmann et al. 2002). Still, strength of hippocampal‐medial parietal lobe connectivity has previously been shown to relate to tau pathology burden in the medial parietal lobe cross‐sectionally (Ziontz et al. 2021; Jacobs et al. 2018), and disruption of hippocampal network connectivity has been proposed as the basis of cognitive deficits in AD (La Joie et al. 2014).

We further showed that the relationship between functional connectivity and tau accumulation was modulated by both baseline tau and global Aβ burden. Aβ has been associated with changes in network connectivity early in disease progression (Giorgio et al. 2024), and modeling of tau spreading in individuals along the AD continuum has suggested that there are both local and remote tau/Aβ interactions that influence the propagation of tau pathology (Lee et al. 2022). Thus, greater cortical Aβ pathology in individuals in our study may reflect changes in connectivity between hippocampus and downstream cortical areas that facilitate the fastest accumulation of tau. We also found that ApoE genotype modulated this relationship, as carriers of the ApoE4 gene exhibited greater tau accumulation in precuneus with greater HC‐PrC functional connectivity and baseline tau pathology. This finding may be related to the underlying influence of ApoE4 genotype on AD pathology, as patterns of tau spread in cortex are spatially linked with ApoE messenger RNA expression (Montal et al. 2022). Further, Aβ burden has been proposed as a mediating factor in the relationship between ApoE4 risk and change in tau pathology over time in nondemented individuals (Steward et al. 2023). It may therefore be that for unimpaired individuals at the earliest stages of tau pathology spread, ApoE4 positivity modulates the relationship between connectivity and tau accumulation via its influence on cortical Aβ burden. Furthermore, given our finding that ApoeE4 status modulated this relationship independently of Aβ, ApoE4 genotype may have additional influence on cellular hyperactivity (Koutsodendris et al. 2023; Nuriel et al. 2017; Ferrari‐Souza et al. 2023), further driving downstream tau pathology spread (Therriault et al. 2020). However, this framework is complicated by our finding that the strongest relationship between functional connectivity and tau accumulation in IT was in fact found in ApoE4 noncarriers. One possible explanation is that because IT is an area of very early neocortical tau accumulation, ApoE4 carriers already demonstrate alterations in network connectivity (Filippini et al. 2009; Cacciaglia et al. 2023) such that greater connectivity is no longer associated with the greatest degree of tau accumulation. This finding also underscores that there are likely multiple interacting factors that influence the extent and distribution of downstream pathology accumulation, and it may be that excitatory changes associated with ApoE genotype (Nuriel et al. 2017; Filippini et al. 2009) outweigh those associated with intrinsic connectivity strength in some pathways. This possibly should be further investigated in larger samples with more ApoE genotypic diversity.

Our finding that HC‐PrC pathology‐connectivity interactions was related to rate of visuospatial memory decline, more so than verbal memory decline, represents a compelling neuropsychological consequence of the modulation of functional connectivity by AD pathology. Both in individuals with lower levels of baseline Aβ and in ApoE4− individuals, we observed that greater functional connectivity strength was associated with slower rates of visuospatial memory decline even with greater high baseline hippocampal tau pathology. This relationship was markedly different at high levels of cortical Aβ and in ApoE4+ individuals, however, where greater hippocampal tau and HC‐PrC was associated with the steepest rates of visuospatial memory decline (Figure 5). This finding comports with the observed relationships between HC‐PrC pathology‐connectivity interaction and tau accumulation, in that the 3‐way interaction between functional connectivity, baseline tau, and baseline Aβ/ApoE4 genotype is associated not only with faster tau pathology accumulation but also faster cognitive decline. Furthermore, It is notable that we observed this relationship to be particularly strong for visuospatial memory decline and HC‐PrC connectivity given the proposed role of medial parietal structures in both episodic memory and visuospatial processing (Ranganath and Ritchey 2012; Bushara et al. 1999). Because we did not observe strong associations between HC‐IT connectivity and tau accumulation in the BACS sample, future work is needed to assess if this connectivity is also related to cognitive decline in domains that rely on temporal structures such as object/face recognition (Ranganath and Ritchey 2012; Peelen and Caramazza 2012). Taken together, the present findings suggest that greater functional connectivity between medial temporal lobe and neocortical regions in the absence of AD pathology is in fact beneficial for cognitive function, but the presence of Aβ and/or ApoE4 genotype may alter the excitatory/inhibitory balance of neural circuits along robust functional connections (Harris et al. 2020; Ranasinghe et al. 2022), leading to the spread of pathological tau protein and subsequent cognitive decline.

This study has a number of limitations that should be considered. First, we are limited in statistical power by the size of the sample with all necessary data types available across both cohorts. Particularly for testing models with 2‐ and 3‐way interactions, this lack of statistical power yielded results that likely would not survive multiple comparisons corrections across all regions of the brain were we to take a less hypothesis‐driven approach for which regions to test. In addition, though the pattern of results was consistent for both BACS and ADNI, individual findings were not always replicated across cohorts, limiting their generalizability to all cognitively unimpaired individuals. This may be related to the inherent noise of resting state functional MRI data. While a compelling tool for examining the relationship between brain connectivity and AD pathology, this modality is often confounded by noise to due to motion and signal dropout in ventral regions of cortex for which we may not have been fully able to adjust. Future work should replicate these findings in better powered samples, and investigate alternative methods for reducing the inherent noise of fMRI data such as the use of partial correlations to compute functional connectivity (Mahadevan et al. 2021). In addition, the integrity of structural connections measured with diffusion MRI may provide further insight into how the structural and functional pathways in the brain provide a roadmap for neuropathologies to propagate, and what other modulating factors have the potential to exacerbate or slow the spread of pathology along these pathways.

## 4 Conclusion

The present study provides evidence that longitudinal tau accumulation can be predicted by the interaction of baseline AD pathology and resting state functional connectivity between vulnerable regions of the brain. Within the framework of networks as both conduits and drivers of neurodegenerative pathology in the aging brain (Vogel et al. 2023), functional connections may be viewed as the pathways along which tau pathology spreads, and factors such as upstream tau pathology, global Aβ burden, and ApoE genotype represent modulating factors that influence the spatiotemporal pattern of future tau spreading. By focusing on cognitively unimpaired individuals, i.e., those in the earliest stages of tau propagation, this study points to characteristics that could help identify which individuals are at greatest risk of progression to AD and could therefore benefit the most from pathology‐lowering interventions.

## 5 Methods

### 5.1 Participants

We included data from 110 cognitively unimpaired (CU) older adults from the Berkeley Aging Cohort Study (BACS) and Alzheimer's Disease Neuroimaging Initiative (ADNI). Demographic information for all participants is shown in Table 1, and demographics for each sample separately is shown in Table S1. All participants had data from 3 T structural MRI, resting state 3 T functional MRI, flortaucipir (FTP) PET scan within 1 year of MRI (*M* = 45 days, SD = 52 days), Aβ PET imaging, and at least two separate FTP PET scans. We excluded 6 participants on the basis of excessive head motion during the rsfMRI scan, defined as > 20% of all volumes being flagged as outliers during artifact detection. Additional inclusion criteria in BACS were 60+ years of age, cognitively normal status Mini Mental State Examination score ≥ 25 and normal neuropsychological examination, defined as within 1.5 SDs of age, education, and sex adjusted norms; no serious neurological, psychiatric, or medical illness, no major contraindications found on MRI or PET, and independent community living status. ADNI participants were all cognitively normal or had subjective memory complaints at time of their rsfMRI scan. This study was approved by the Institutional Review Boards of the University of California, Berkeley, and the Lawrence Berkeley National Laboratory (LBNL). All participants provided written informed consent. The final sample of 110 cognitively unimpaired individuals included 67 cognitively normal BACS participants and 43 cognitively unimpaired ADNI participants.

### 5.2 MRI Acquisition and Processing

In BACS, structural and functional MRI data were acquired on a 3 T TIM/Trio scanner (Siemens Medical System, software version B17A; Siemens, Erlangen, Germany) using a 32‐channel head coil. A T1‐weighted whole brain magnetization prepared rapid gradient echo (MPRAGE) image was acquired for each subject (voxel size = 1 mm isotropic, TR = 2300 ms, TE = 2.98 ms, matrix = 256 × 240 × 160, FOV = 256 × 240 × 160 mm^3^, sagittal plane, 160 slices, 5 min acquisition time). Resting state functional MRI was then acquired using T2*‐weighted echo planar imaging (EPI, voxel size = 2.6 mm isotropic, TR = 1.067 s, TE = 31.2 ms, FA = 45, matrix 80 × 80, FOV = 210 mm, sagittal plane, 300 volumes, anterior to posterior phase encoding, ascending acquisition, 5.3 min acquisition time). During resting state acquisition, participants were told to remain awake with eyes open and focused on a white asterisk displayed on a black background.

In ADNI, structural and functional MRI data were acquired on 3 T scanners with a standardized protocol across all sites (Siemens Medical Solutions, Siemens, Erlangen, Germany). A T1‐weighted whole brain magnetization prepared rapid gradient echo (MPRAGE) image was acquired for each subject (voxel size = 1 mm isotropic, TR = 2300 ms, TE = min full echo, matrix = 208 × 240 × 256, FOV = 208 × 240 × 256 mm^3^, sagittal plane, 256 slices, 6 min acquisition time). Resting state functional MRI was then acquired using EPI‐BOLD (voxel size = 2.5 mm isotropic, TR = 0.607 s, TE = 30 ms, FA = 53, matrix 220 × 220, FOV = 160 mm, sagittal plane, 976 volumes, anterior to posterior phase encoding, ascending acquisition, 10 min acquisition time, further details at https://adni.loni.usc.edu/wp‐content/uploads/2017/07/ADNI3‐MRI‐protocols.pdf). Structural T1‐weighted images were processed using Statistical Parametric Mapping (SPM12; https://www.fil.ion.ucl.ac.uk/spm/software/spm12/). Images were first segmented into gray matter, white matter, and CSF components in native space. Native space T1 images were segmented with Freesurfer v.5.3.0 (https://surfer.nmr.mgh.harvard.edu/) using the Desikan–Killany atlas parcellation (Desikan et al. 2006).

Resting state fMRI images from BACS and ADNI were preprocessed using a nearly identical SPM12 pipeline, with minimal differences to account for different acquisition parameters. Slice time correction was applied in BACS to adjust for differences in acquisition time for each brain volume, but this was not done in ADNI because of the low TR resting state data. In both cohorts, all EPIs were realigned to the first acquired EPI, and translation and rotation realignment parameters were output. Each EPI was next coregistered to each individual's native space T1 image, and these native space rsfMRI scans were used to extract the time series of each ROI in seed‐to‐seed functional connectivity analyses.

### 5.3 Functional Connectivity Analyses

ROI‐to‐ROI functional connectivity was assessed using the CONN functional connectivity toolbox (version 17e) (Whitfield‐Gabrieli and Nieto‐Castanon 2012) implemented in MATLAB version 2021a (The Mathworks Inc., Natick, MA). ART motion detection (https://www.nitrc.org/projects/artifact_detect/) was first performed to identify volumes of high motion, using a movement threshold of > 0.5 mm/TR and a global intensity z‐score of 3. Outlier volumes were flagged and included as spike regressors during denoising; no volumes were discarded. Denoising was performed with translation and rotation realignment parameters and their first‐order derivatives, as well as anatomical CompCor (first five components of time series signal from white matter and CSF). A band pass filter of 0.008–0.1 Hz and linear detrending were applied to the residual time‐series. For each individual, denoised time series were extracted for 87 ROIs from the Desikan–Killiany atlas (Desikan et al. 2006). Each of these time series were then correlated (Pearson's r) and transformed (Fisher r to z). As a final step to compare functional connectivity between the BACS and ADNI samples, these values were z‐scored within each sample using the mean and standard deviation of Aβ− individuals for each ROI‐ROI connection.

We examined pathology‐connectivity interactions as the 3‐way interaction between functional connectivity, tau pathology, and Aβ pathology burden. We conducted seed‐target connectivity analyses with linear regression models to assess the relationship between rate of tau pathology accumulation in the downstream target region (either precuneus or inferior temporal cortex) and the resting state functional connectivity strength between the seed region (hippocampus) and this target region, modulated by baseline tau and either baseline Aβ or ApoE4 status. The seed‐target analysis was carried out with linear regression models and included all 2‐way interactions and main effects related to the 3‐way interaction of interest.

### 5.4 PET Acquisition and Processing

In BACS, PET was acquired for all participants at the Lawrence Berkeley National Laboratory (LBNL). Tau burden was assessed with ^18^F‐Flortaucipir (FTP) synthesized at the Biomedical Isotope Facility at LBNL as previously described (Schöll et al. 2016). Data were collected on a Biograph TruePoint 6 scanner (Siemens Inc) 75–115 min postinjection in listmode. Data were then binned into 4 × 5 min frames from 80 to 100 min postinjection. CT scans were performed before the start of each emission acquisition. In ADNI, PET data were collected using a standardized protocol of six 5‐min time windows 75 to 105 min after injection. All PET images were reconstructed using an ordered subset expectation maximization algorithm, with attenuation correction, scatter correction, and smoothing with an 8 mm Gaussian kernel.

Processing of FTP images was carried out in SPM12 and Freesurfer. Images were realigned, averaged, and coregistered to 3 T structural MRIs. Standardized uptake value ratio (SUVR) images were calculated by averaging mean tracer uptake over the 80–100 min data and normalized with an inferior cerebellar gray reference region. The mean SUVR of each ROI (structural MRI FreeSurfer segmentation) was extracted from the native space images. Because hippocampal FTP signal is often thought to have low validity due to contamination from off‐target choroid plexus signal (Baker et al. 2019), regional SUVRs were partial volume corrected (PVC) using a modified Geometric Transfer Matrix approach (Rousset, Ma, and Evans 1998) as previously described (Baker, Maass, and Jagust 2017) to minimize spillover signals between adjacent regions. Additionally, all analyses with hippocampal FTP included choroid plexus PVC SUVR as a covariate. The regional rate of tau pathology accumulation was computed using a longitudinal PET processing pipeline that defines a midpoint average MRI from all baseline and follow‐up scans to coregister PET images (Harrison et al. 2019). Tau slopes were extracted from linear mixed effects models with time from baseline as the only predictor and random effects of slope and intercept. All available FTP PET scans were included in each individual's slope calculation, and SUVR values were normalized to a hemispheric eroded white matter reference region prior to modeling to reduce variability over time. Slopes were computed for the 110 individuals in our final sample from a total of 309 FTP PET scans (*M* = 2.81, SD = 0.88). Defining rsfMRI scan as baseline, 72.7% of FTP scans were prospective and 27.2% of FTP scans were retrospective across all individuals. Voxelwise tau slope images were computed using all available FTP PET scans for each individual, with slope values from standard linear regression extracted for each voxel in the brain.

IN BACS, Aβ burden was assessed using ^11^C‐Pittsburgh Compound B (PiB), also synthesized at the Biomedical Isotope Facility at LBNL (Mathis et al. 2009). Data were collected on the Biograph scanner across 35 dynamic frames for 90 min postinjection and subsequently binned into 35 frames (4 × 15, 8 × 30, 9 × 60, 2 × 180, 10 × 300, and 2 × 600 s), and a CT scan was performed. All PET images were reconstructed using an ordered subset expectation maximization algorithm, with attenuation correction, scatter correction, and smoothing with a 4 mm Gaussian kernel. An average of frames within the first 20 min was used to calculate the transformation matrix to coregister the PiB images to the participants' 3 T structural MRI; this transformation matrix was then applied to all PiB frames. Distribution volume ratio (DVR) images were calculated with Logan graphical analysis over 35–90 min data and normalized to a whole cerebellar gray reference region (Logan 2000; Price et al. 2005). Global Aβ was calculated across cortical FreeSurfer ROIs as previously described (Mormino et al. 2012), and a threshold of DVR > 1.065 was used categorize participants as Aβ‐positive or Aβ‐negative in BACS. In ADNI, Aβ burden was assessed using either florbetapir (FBP) or florbetaben (FBB), with an SUVR cutoff of 1.08 and 1.11, respectively, used to categorize Aβ‐positive and Aβ‐negative participants. Finally, Aβ PET measures were converted to Centiloids using standard conversion formulae (Giorgio et al. 2022).

### 5.5 Neuropsychological Measures

The visuospatial and verbal memory composites in BACS were constructed from longitudinal cognitive data taken from a standard neuropsychological battery. Longitudinal cognitive data included a total of 541 observations for the 67 individuals in BACS (*M* = 8.1, SD = 3.3 observations per individual). Participants had an average of 4.4 cognitive time points prior to baseline MRI (SD = 2.3), and an average of 3.7 cognitive time points after baseline MRI (SD = 1.8). Visuospatial memory was defined as a composite of Visual Reproduction I and II total recall scores. Verbal memory was defined as a composite of California Verbal Learning Test short‐ and long‐delay‐free recall components. All scores were z‐scored using the mean and standard deviation of each test from baseline, defined as the cognitive time point closest to each participant's rsfMRI scan. We computed the rate of cognitive decline for each individual by extracting slopes from linear mixed effects models with time from baseline as the only predictor and random effects of slope and intercept.

### 5.6 Statistical Analyses

All terms used in linear models with interactions were mean‐centered. All linear mixed effects modeling of regional tau slopes, pathology‐connectivity interactions, and cognitive decline in BACS were conducted using {nlme} in R. Standard linear regression was carried out with {lm} in R.

### 5.7 Data Visualization

Surface renderings of voxelwise tau slope images were visualized using the BrainNet Viewer toolbox (http://www.nitrc.org/projects/bnv/) in MATLAB Version 23.a. Ball‐and‐stick plots to visualize connectivity were created using the *nilearn* glassbrain plotting function in python (https://nilearn.github.io/dev/modules/generated/nilearn.plotting.plot_glass_brain.html). Scatter plots were created using the {ggplot2} package in R version 4.2.0 to visualize effects from linear regression models. The {effects} package in R was used to visualize interaction effects from linear regression models. The *seaborn.regplot* was used for scatter plots to visualize the correlation between predicted and observed outcomes from leave‐one‐out cross‐validation.

## Author Contributions

Conceptualization: J.Z., W.J.J. Data curation: J.Z., T.M.H., C.F. Formal analysis: J.Z. Funding acquisition: J.Z., W.J.J. Methodology: J.Z., T.M.H., J.J.G., F.H., W.J.J. Project administration: W.J.J., Resources: W.J.J. Software: J.Z., C.F. Visualization: J.Z., C.F., Writing: J.Z., W.J.J. Review and Editing – all authors.

## Disclosure

Avid Radiopharmaceuticals enabled the use of and the [18] Flortaucipir tracer but did not provide direct funding and was not involved in data analysis or interpretation. W.J.J. consults for Lilly, Eisai, Biogen, and Bioclinica.

## Ethics Statement

This study was conducted following the ethical guidelines of the IRB of the University of California, Berkeley, and Lawrence Berkeley National Laboratory. Informed consent was obtained from all participants from the Berkeley Aging Cohort Study as well as the Alzheimer's Disease Neuroimaging Initiative.

## Supporting information


Data S1.

## Data Availability

All analyses in this study were conducted using a combination of software and custom code utilizing standard neuroimaging and statistical packages, detailed in the Methods section. All code and BACS data is available upon request, and ADNI data is publicly available at https://adni.loni.usc.edu/.
