# Supplementary material for: Connectivity, Pathology, and ApoE4 Interactions Predict Longitudinal Tau Spatial Progression and Memory
Source: Hum Brain Mapp. 2024 Dec 9;45(17):e70083. doi: 10.1002/hbm.70083 (PMC11626484; doi:10.1002/hbm.70083)
Supplement: Supplementary file 1 — Data S1. [file HBM-45-e70083-s001.docx]

***Supplementary Data***

|  | ***BACS CU (n=67)*** | ***ADNI CU (n=43)*** | ***p-value*** |
| --- | --- | --- | --- |
|  | *Mean (SD)* | | |
| *Age (years)* | *77.1 (5.8)* | *75.4 (7.3)* | *0.22* |
| *Centiloids* | *23.7 (32.6)* | *34.6 (45.9)* | *0.18* |
| *Meta ROI tau slope (SUVR/year)* | *0.02 (0.01)* | *0.02 (0.01)* | *0.20* |
| *rsfMRI-tau interval (days)* | *54.2 (54.3)* | *30.7 (45)* | ***0.02*** |
| *Sex (female)* | *38 (57%)* | *29 (67%)* | *0.36* |
| *Aβ+* | *33 (49%)* | *19 (44%)* | *0.75* |
| *APO𝜀4+* | *22 (34%)* | *13 (31%)* | *0.88* |

**Table S1.** *Demographics of separated BACS and ADNI cohorts of cognitively unimpaired (CU) older adults.*

**
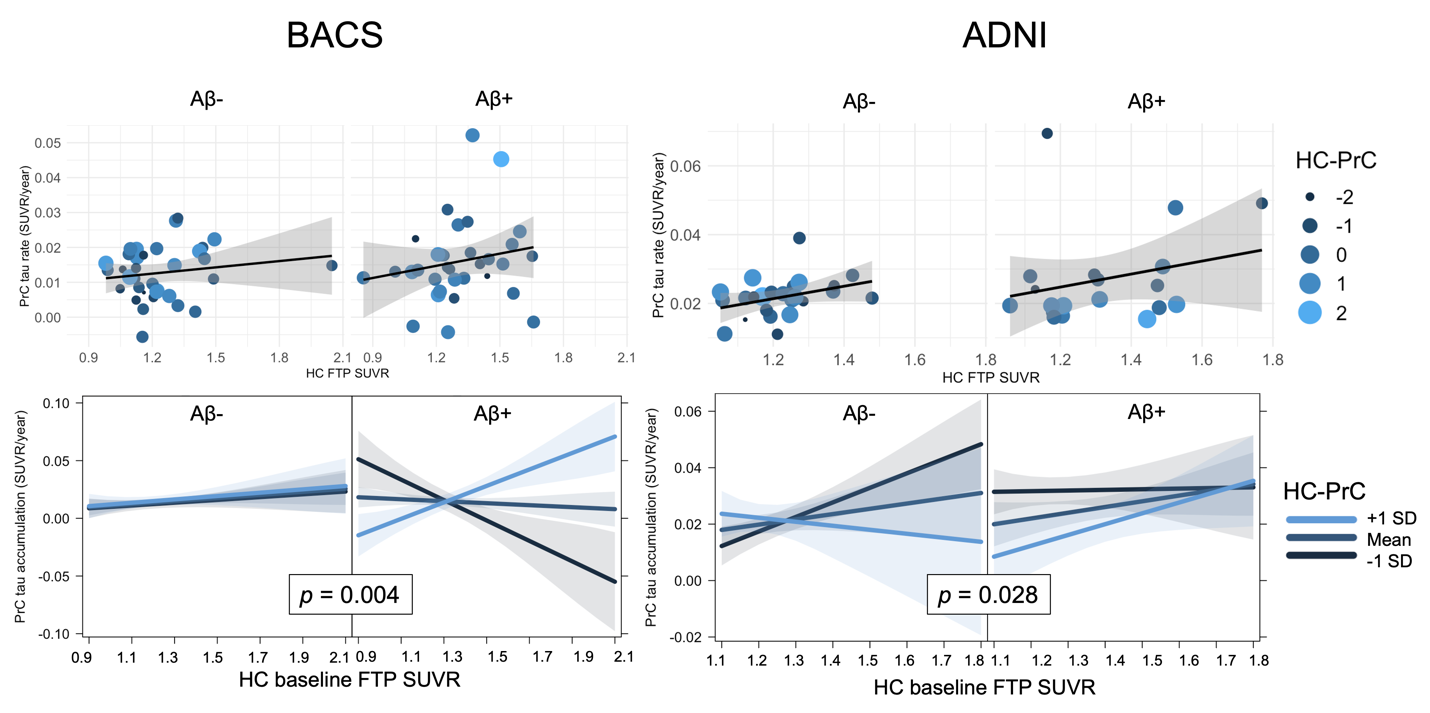
**

**Figure S1.** *Pathology- connectivity interaction is associated with precuneus tau accumulation in separate cohorts.* Lines represent predicted association for 3 different levels of HC-PrC connectivity. Panels visualize predicted associations at mean Centiloid value of Aβ- (*left*) and Aβ+ (*right)* participants.

**
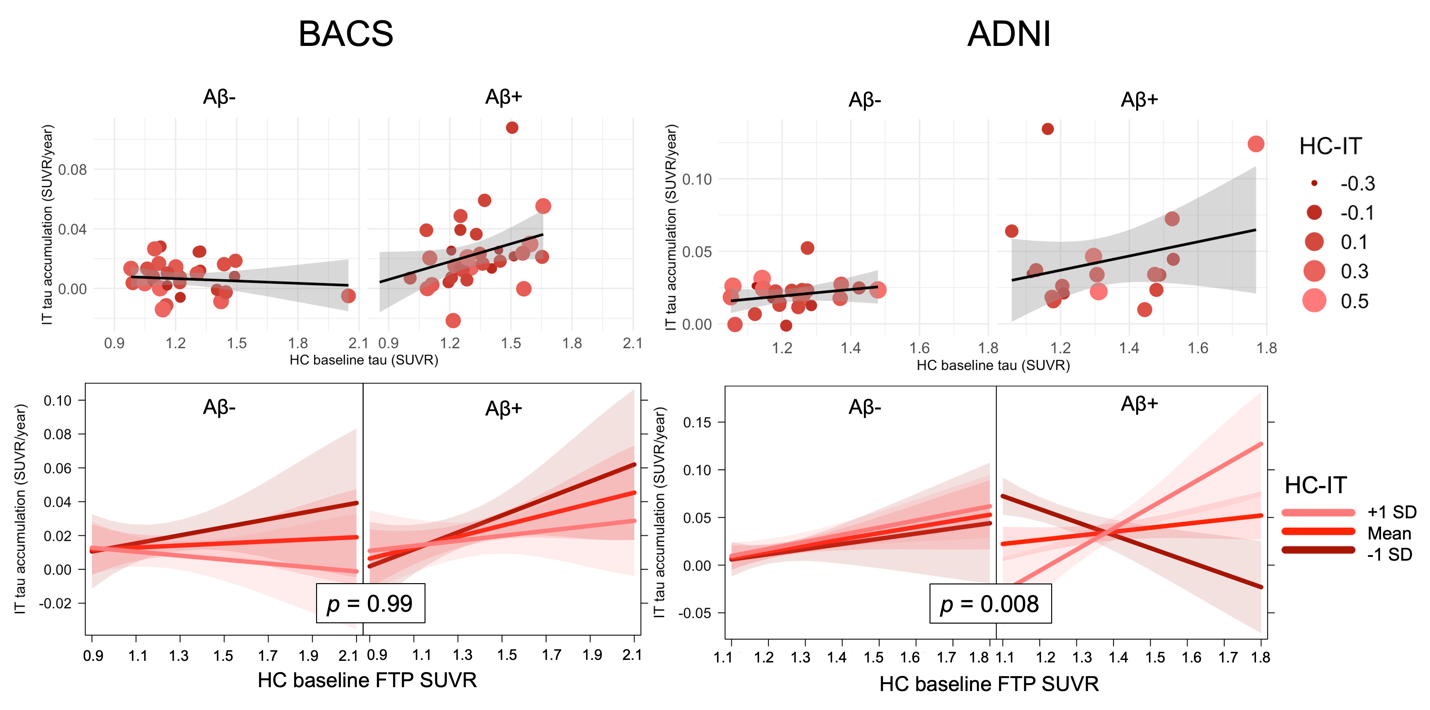
**

**Figure S2.** *Pathology-connectivity interaction is associated with inferior temporal tau accumulation in separate cohorts*. Lines represent predicted association for 3 different levels of HC-PrC connectivity. Panels visualize predicted associations at mean Centiloid value of Aβ- (*left*) and Aβ+ (*right)* participants.

**
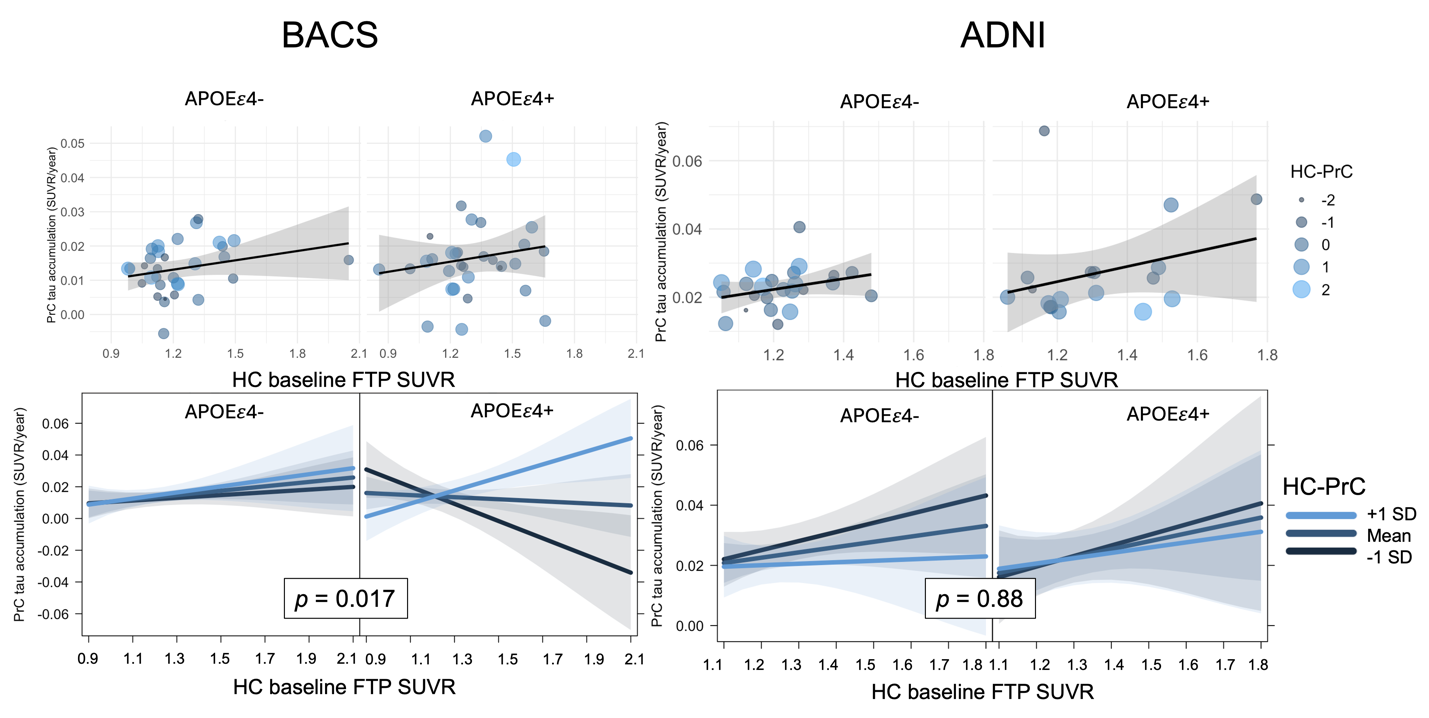
**

**Figure S3.** *Connectivity modulated by APOEε4 genotype is associated with precuneus tau accumulation in separate cohorts.* Lines represent predicted association for 3 different levels of functional connectivity. Panels visualize predicted associations for *APOEε4-* (*left*) and *APOEε4+* (*right)* participants.

**
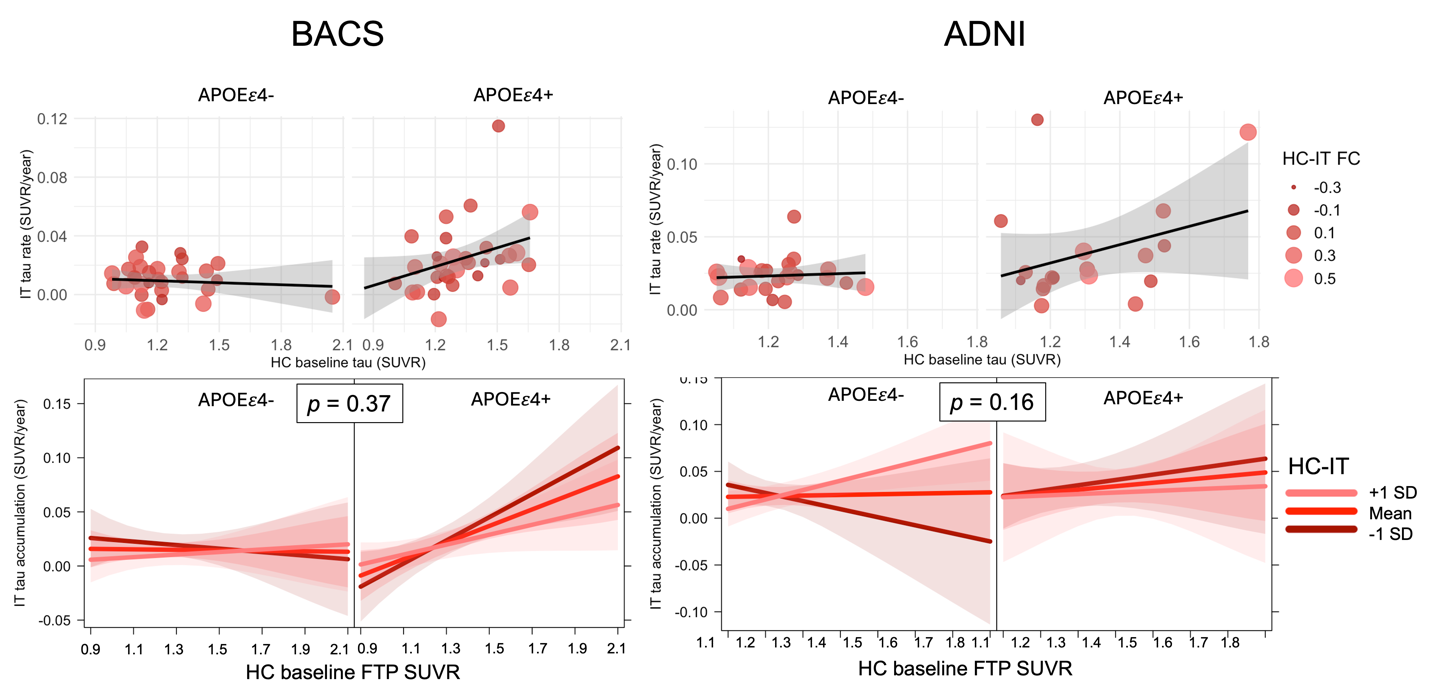
**

**Figure S4.** *Connectivity modulated by APOEε4 genotype is associated with inferior temporal tau accumulation in separate cohorts.* Lines represent predicted association for 3 different levels of functional connectivity. Panels visualize predicted associations for *APOEε4-* (*left*) and *APOEε4+* (*right)* participants.

**
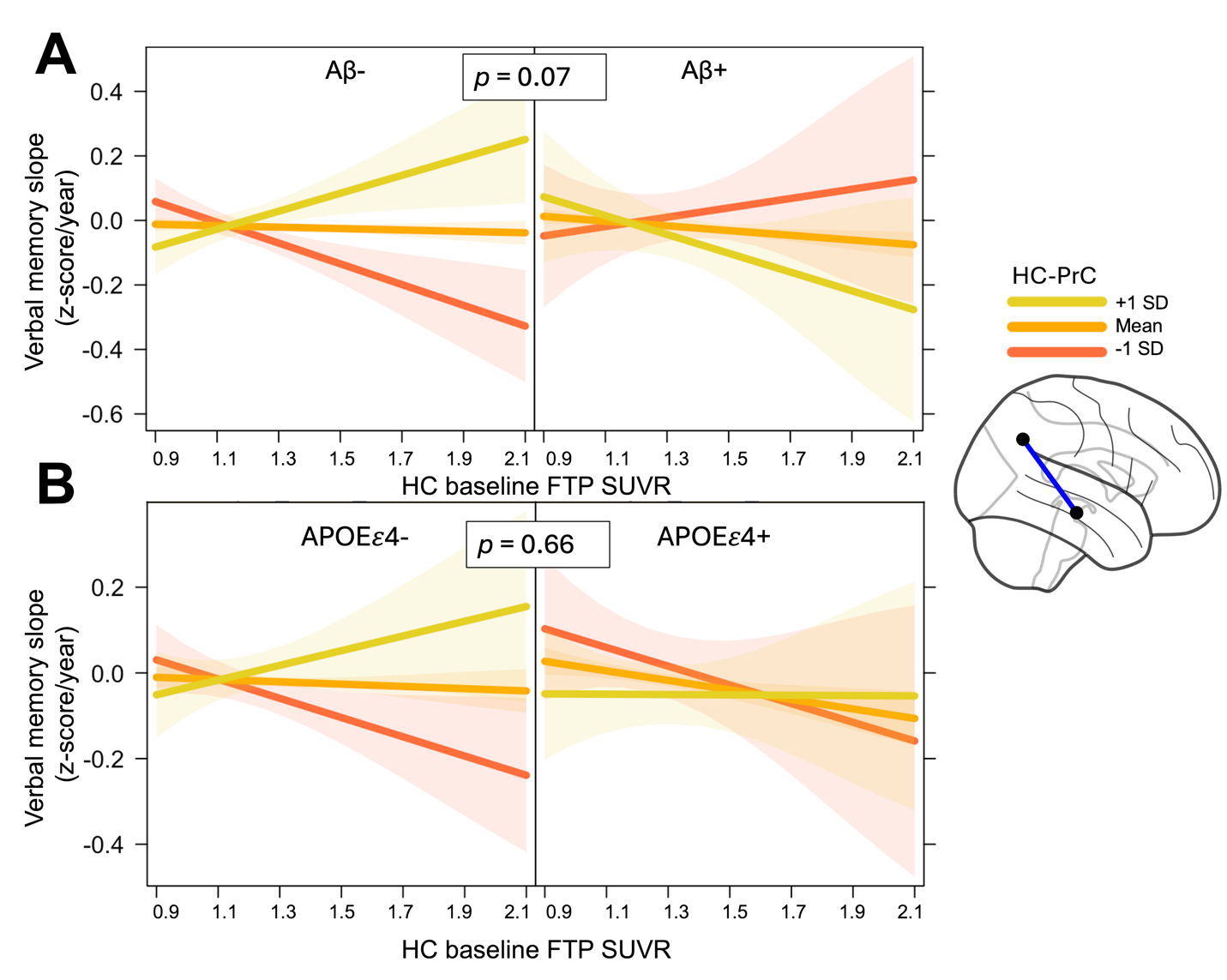
**

**Figure S5.** *Association of HC-PrC pathology-connectivity interaction and verbal memory decline.* (A) Visualization of 3-way interaction between hippocampus-precuneus functional connectivity (HC-PrC), baseline HC tau, and Centiloids. (B) Visualization of 3-way interaction between hippocampus-precuneus functional connectivity (HC-PrC), baseline HC tau, and *APOEε4* status. Lines represent predicted association for 3 different levels of functional connectivity. Panels visualize predicted associations for *APOEε4-* (*left*) and *APOEε4+* (*right)* participants.
